# Effectiveness of Educational Poster on Knowledge of Emergency Management of Dental Trauma–Part 1. Cluster Randomised Controlled Trial for Primary and Secondary School Teachers

**DOI:** 10.1371/journal.pone.0074833

**Published:** 2013-09-11

**Authors:** Cecilia Young, Kin Yau Wong, Lim K. Cheung

**Affiliations:** 1 Private Practice, Hong Kong; 2 Department of Biostatistics, University of North Carolina at Chapel Hill, Chapel Hill, North Carolina, United States of America; 3 Chair Professor, Oral and Maxillofacial Surgery, Faculty of Dentistry, The University of Hong Kong, Hong Kong; University of South Australia, Australia

## Abstract

**Objective:**

To investigate the effectiveness of educational posters in improving the knowledge level of primary and secondary school teachers regarding emergency management of dental trauma.

**Methods:**

A cluster randomised controlled trial was conducted. 32 schools with a total of 515 teachers were randomised into intervention (poster) and control groups at the school level. Teachers’ baseline levels of knowledge about dental trauma were obtained by using a questionnaire. Posters containing information on dental trauma management were displayed in the school medical room, the common room used by staff, and on a notice board for 2 weeks in each school of the intervention group; in the control group, no posters were displayed. Teachers in both groups completed the questionnaire after 2 weeks.

**Results:**

The teachers in the intervention schools (where posters were displayed for 2 weeks) showed statistically significant improvement in scores in cases where they had not previously learned about dental emergencies from sources other than first aid training, with an average score increase of 2.6656 (score range of questionnaire, −13 to 9; p-value <0.0001).

**Conclusion:**

Educational posters on the management of dental trauma can significantly improve the level of knowledge of primary and secondary school teachers in Hong Kong.

KClinicalTrials.com HKCTR-1307

ClinicalTrials.gov: NCT01707355

## Introduction

Emergency management of traumatic dental injuries is of key importance, as the prognosis of traumatized teeth, especially from avulsion, depends on prompt management [1]. School is one of the most common places where dental injuries occur [2]–[5]. Teachers can help to improve the prognosis of these injuries if they know how to manage at the site of injury. However, many studies have revealed that teachers lack this essential knowledge [6]–[11]. The authors suggested health promoters to launch educational campaigns for teachers.

A literature search of studies on education in traumatic dental injuries took place before the study and was finalized on January 29, 2013 (search terms included “education*” or “intervention” or “promotion” or “campaign” or “knowledge” or “lecture” or “seminar” or “leaflet” or “pamphlet” or “poster”) and (“dental trauma” or “traumatic dental injur*” or “dental injur*”). Only 13 papers related to education of teachers in the management of traumatic dental injuries were found on PubMed, Ovid, Web of Science, and the Cochrane Central Register of Controlled Trials through this search [12]–[24].

In the mentioned studies, the methods used for promotion were seminars or lectures [12]–[17], [22]–[24], leaflets or pamphlets [16]–[20], banners [20], and posters [21]. The subjects were teachers or school staff [12], [13], [15]–[18], [20], [21], [24], parents [19], army [14] and other professionals [15], [22], [23]. Most of these studies showed that the intervention improved the level of subjects’ knowledge [12]–[16], [18]–[24]; in one exception to this, Kahabuka et al., using children’s self-care as an end point, concluded that seminars or leaflets to teachers were not good enough to promote children’s self-care after dental injuries [17].

Lieger et al. investigated the effect of educational posters on the knowledge of management of dental injuries over a 5-year period [21]. They sent educational posters to 100 schools in Canton of Bern in 2001, and followed up with 1000 questionnaires to these schools in 2006. They also sent 1000 questionnaires to another school group in which no poster campaign was launched, Cantons of Lucerne and St. Gallen (also in 2006). They compared the level of knowledge of teachers in the two groups by the results of the questionnaires, which showed that educational posters effectively improved the level of knowledge on dental trauma management.

A poster campaign is relatively easy to implement because of its low cost. Such a campaign does not require gathering teachers at a particular time (as would be necessary for seminars). Asking principals to display the posters is also a simple request. The present cluster randomised controlled trial investigated the effectiveness of educational posters about traumatic dental injuries in improving the knowledge level of primary and secondary school teachers. We adopted the cluster design because the intervention was conducted on the school level instead of on the level of the individual teacher. Teachers in the same school read the same posters and may discuss these posters with each other, exerting influence in the process. Teachers in the same school may also have some special characteristics, for example, a particular interest in health information. In order to minimize contamination of results, school was considered as the unit of randomisation.

## Materials and Methods

### Ethical Approval

The research project was approved by the Institutional Review Board of the University of Hong Kong and Hospital Authority Hong Kong West Cluster. (HKCTR-1307, ClinicalTrials.gov:NCT01707355). The protocol for this trial and supporting CONSORT checklist are available as supporting information; see Checklist S1 and Protocol S1.

### Design

#### Subjects

The target group of this study included teachers working in primary (US grades 1–6) and secondary (US grades 7–12 plus 1 year) schools in Hong Kong who could read Chinese or English. We recruited the primary or secondary schools as clusters.

#### Questionnaire

A questionnaire from a related survey about knowledge of dental trauma among the same target group was used [11]. The questionnaire was written in Chinese and English and was composed of 2 sections with a total of 14 questions. Questions in the first section collected basic demographic information and asked whether the respondents had received formal first aid training or had acquired dental trauma information from other sources. Respondents were also asked whether they considered themselves able to distinguish deciduous teeth from permanent teeth. The questions in the second section related to management of traumatic dental injuries. The questionnaire was pilot-tested on 81 teachers in order to determine optimal length and ascertain whether targeted recipients could comprehend the questions; questions were pre-tested in this manner before adoption. The face validity was established by expert opinion. The test-retest reliability test also indicated that the scores of the first and second questionnaires were positively correlated. The marking scheme is presented in the footnote of Table 1.

**Table 1:** Scores of both questionnaires of both groups.

	Intervention group (*n* = 196) Number (%)		Control group (*n* = 212) Number (%)	
	Baseline Score	Q2 Score	Baseline Score	Q2 Score
**Q9** Place for treatment				
Correct	60 (30.6)	98 (50.0)	62 (29.2)	57 (26.9)
Incorrect	124 (63.3)	94 (48.0)	142 (67.0)	149 (70.3)
Do not know	12 (6.1)	4 (2.0)	8 (3.8)	6 (2.8)
**Q10** Time for treatment				
Correct	138 (70.4)	144 (73.5)	159 (75.0)	155 (73.1)
Incorrect	44 (22.4)	47 (24.0)	45 (21.2)	48 (22.6)
Do not know	14 (7.1)	5 (2.6)	8 (3.8)	9 (4.2)
**Q11** Management of fractured teeth				
Correct	66 (33.7)	114 (58.2)	79 (37.3)	84 (39.6)
Incorrect	96 (49.0)	69 (35.2)	103 (48.6)	102 (48.1)
Do not know	34 (17.3)	13 (6.6)	30 (14.2)	26 (12.3)
**Q12** Management of displaced teeth				
Correct	45 (23.0)	96 (49.0)	42 (19.8)	57 (26.9)
Incorrect	128 (65.3)	86 (43.9)	145 (68.4)	128 (60.4)
Do not know	23 (11.7)	14 (7.1)	25 (11.8)	27 (12.7)
**Q13i** Management of avulsed baby teeth				
Correct	133 (67.9)	138 (70.4)	166 (78.3)	153 (72.2)
Incorrect	12 (6.1)	27 (13.8)	6 (2.8)	12 (5.7)
Do not know	51 (26.0)	31 (15.8)	40 (18.9)	47 (22.2)
**Q13ii** Management of avulsed permanent teeth				
Correct	37 (18.9)	80 (40.8)	37 (17.5)	39 (18.4)
Incorrect	98 (50.0)	82 (41.8)	107 (50.5)	90 (42.5)
Do not know	61 (31.1)	34 (17.3)	68 (32.1)	83 (39.2)
**Q14** Mediums for storage of avulsed teeth				
	Mean = −0.122	Mean = 0.918	Mean = −0.198	Mean = −0.170
	Std. Dev. = 1.295	Std. Dev. = 1.621	Std. Dev. = 1.341	Std. Dev. = 1.390
**Total Score**				
	Mean = −0.240	Mean = 2.270	Mean = −0.212	Mean = −0.094
	Std. Dev. = 2.775	Std. Dev. = 4.011	Std. Dev. = 2.868	Std. Dev. = 3.054
	Score Change = 2.510		Score Change = 0.118	
	Std. Dev. = 3.776		Std. Dev. = 2.277	

For Questions 9–13, 1 mark for each correct answer, 0 for don’t know, −1 if it is wrong or any wrong answer if more than 1 was chosen. (−6 to 6).

For question 14, 1 for each correct answer, 0 for don’t know, −1 for each wrong answer (−7 to 3).

Range of total scores for the whole questionnaire: −13 to 9.

#### Poster

Colorful educational poster of A3 size (roughly 30 by 42 cm) with pictures was designed (see Chinese Educational Poster S1 and English Educational Poster S1). One side of the poster was in Chinese and the other side in English. The information was constructed by the authors with reference to two publications [11], [21]. The information included the following: (a) a permanent tooth should be placed back to the socket immediately, but a primary tooth should not be; (b) children start to have 1–28 permanent teeth between the ages of 5 and 12 years; (c) a person should keep calm and stop the bleeding when someone sustains dental traumatic injuries; and (d) lastly, the immediate management procedures for fracture, mobility, displacement, and avulsion.

### Pilot Study

2 schools, one of which was randomly selected to have the poster display (the other did not), were invited to join the pilot study. The first set of questionnaires was sent to both schools; the teachers in charge distributed the hard copies of questionnaire to participating teachers. All participating teachers filled out the first questionnaire and returned to the teachers in charge, who were responsible for returning the completed questionnaires back to the investigator in 1 week.

Three copies of the same educational poster were placed inside a large sealed envelope with instructions included and mailed to the intervention school. The teacher in charge for each school displayed the educational posters in the following venues: a) the medical room; b) the staff common room; and c) any location in the school that had the main purpose of being a “message board” for teachers. The control school did not receive a poster.

After 2 weeks, the posters were removed by the teacher in charge. A second set of questionnaires was then sent to both schools for distribution. The teachers were asked to complete the questionnaires and return them to the study secretary in 1 week using prepaid envelopes.

This process was smooth; the teachers in charge followed all the instructions and no negative feedback from teachers was received. The variance of the score improvement was estimated for sample size calculation.

### Main Study

#### Sample size calculation

In order to demonstrate a difference in score change of 2 marks (range of total score, −13 to 9; variance, 14) between the intervention group and the control group with a power of 90% and a statistical significance of 5%, 74 individuals are needed for each group under simple random sampling. The variance of the score improvement (i.e.14) is estimated from the pilot study. To account for the cluster design, we assume an intra-cluster correlation (ICC) of 0.05. No published data on ICC under this setting can be found. However, in general practice studies, values for ICC are generally between 0.01 and 0.05 [25]. With no less than 10 teachers recruited per school, the adjusted sample size is 11 schools per group. To allow for potential dropouts, we aimed to recruit extra 30% individuals per group, yielding a total of 15 schools per group. With that number of schools, we would need at least 150 teachers per group.

#### Recruitment

The Education Bureau provided a list of primary and secondary schools upon request. There were 678 primary and 663 secondary schools in Hong Kong, giving a pool of 1341 schools. Special schools for students with intellectual disabilities were included in these 1341 schools. Invitation letters with school consent forms and individual teacher consent forms were sent to lots of 50 schools starting on March 1, 2011. The contact information of the principal investigator was given in the invitation letter. 32 schools with a total of 515 teachers completed both school and individual teacher consent forms after 600 invitation letters were sent. Not all the teachers from any given participating school joined the study; only those who signed the consent form for individual teachers were recruited. The name and telephone number of the teachers in charge of this study were written in the school consent form.

Some schools did not provide the minimum of 10 participating teachers while some schools contained more than 10 participating teachers; after consideration of the variation of the cluster size, the required power was still achieved.

The cluster randomised controlled trial was conducted in mid May 2011. No educational campaign regarding dental trauma targeting primary and secondary school teachers had been implemented in Hong Kong before.

#### Randomisation and masking

Randomisation was performed after both the school and individual consent forms were returned during the recruitment process. The schools were randomised to the intervention group and the control group with school being the unit of randomisation using sealed envelopes. The secretary wrote the words “intervention group” and “control group” on two pieces of paper separately and then put these pieces of paper into two envelopes. She labelled the sealed envelope of the intervention group as group A and of the control group as group B. She also verified that the envelope was opaque enough that words could not be seen through its surface. An independent person who did not know the details of this study was invited to assist with the randomisation. The secretary created 32 labels (1 to 32) on separate sheets of paper representing 32 schools according to the date of consent forms being returned. Each paper was folded twice and put in an envelope and checked to ensure that the number could not be seen through any of the 32 white envelopes and then put inside a box.

An independent person blinded to the identity of group A and group B drew one envelope for group A and then one for group B alternatively until all the envelopes were drawn. The secretary then opened the envelopes and listed the results of the randomisation.

#### Implementation of the trial

The first set of questionnaires was sent to both groups; the teacher in charge of each school distributed the hard copies of the questionnaire to participating teachers. All participating teachers filled out the first questionnaires and returned them to the teacher in charge, who was responsible for sending the completed questionnaires back to the investigator in 1 week.

Three copies of the same educational poster were placed inside a large sealed envelope with instructions included and mailed to the intervention schools. The teacher in charge for each school displayed the educational posters in the following venues: a) the medical room; b) the staff common room; and c) any location in the school that had the main purpose of being a “message board” for teachers; control group did not receive posters.

After 2 weeks, the posters were removed by the teachers in charge. A second set of questionnaires was then distributed to school from both the intervention and control groups. Teachers were asked to complete the questionnaires and return them to the study secretary in 1 week using prepaid envelopes. Educational posters were mailed to the control group after completion of the study.

#### Withdrawal of teachers from the study

Participating schools or individual teachers could withdraw from the study at any time as mentioned in the consent forms.

#### Data processing

The data were processed to show whether the educational posters were effective in improving the knowledge of teachers. Investigators, the data entry staff and the statistician were blinded to the group randomisation. The statistician was instructed to analyse the results of group A and group B according to the designed method in the protocol. After the adoption of the whole statistical report and preparation of the draft report, the study secretary informed the principal investigator that group A was the intervention group. At that time, the principal investigator changed all the wording as needed in the study materials from “group A” to “intervention group” and “group B” to “control group”.

#### Data analysis

Individual analysis was performed, as our objective and outcome measures pertained to individual level. To investigate the effects of the intervention, along with some baseline information, on the degree to which individual teachers experienced an increase of knowledge related to dental injuries, a multiple linear regression on the score difference between the two questionnaires was conducted. To account for the effect of clustering, a random effect term was included. There was a school-specific random intercept for each school, which had an additive effect on the total score. The school-specific random effect was normally distributed with zero mean and unknown variance.

To select the most appropriate model, a backward elimination method was adopted [26]. It started with inclusion of all covariates in the model: group (intervention/control), the score of the first questionnaire, gender, age, teaching experience, school (primary, secondary, special), first-aid, dental education in first aid, confidence in distinguishing deciduous and permanent teeth, and acquisition of dental injury information from other sources. As the intervention effects possibly differed for people with different backgrounds, the interaction terms between group and each of the other variables were also included in the regression equation. The covariate associated with the highest p-value was eliminated in each interaction until all p-values were smaller than a threshold value of 0.1.

The thresholds of all the statistical tests were set at the 5% level of significance. The statistical analyses were performed using computer software (JMP version 9.0.0, SAS Institute Inc., USA).

## Results

**Figure 1:**
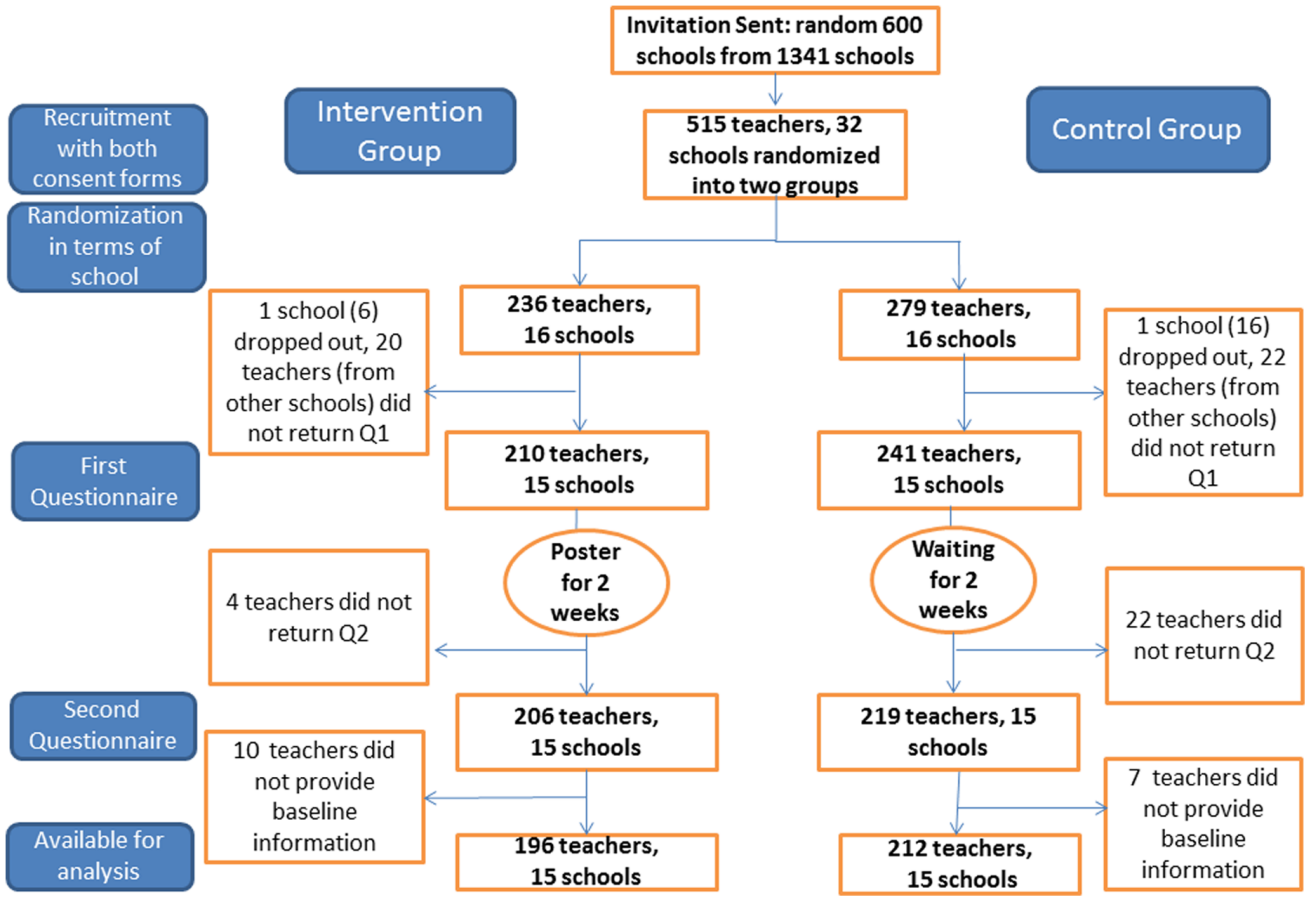
Flow Chart of Participants.

There was no harm or unintended effect reported by participating teachers directly or through the teachers in charge during and after the trial. There were 196 individuals (15 schools) in the intervention group and 212 individuals (15 schools) in the control group (Fig 1). The basic information for both groups on the school level and the individual level are listed in Table 2. Statistical tests to compare scores at baseline were not conducted [27].

**Table 2:** Demographic information and characteristics of both groups on the cluster level and the individual level.

School Level			
	Intervention group (*n* = 15) Number (%)		Control group (*n* = 15) Number (%)
**Cluster Size**	Mean = 13.1		Mean = 14.1
	Median = 10		Median = 12
	Min = 1 Max = 37		Min = 4 Max = 39
**School Type**			
Primary School	4 (26.7)		6 (40.0)
Secondary School	7 (46.7)		5 (33.3)
Include both primary and secondary sections	0 (0.0)		1 (6.7)
Special School	4 (26.7)		3 (20.0)
**Teacher Level**			
	Intervention group (*n* = 196) Number (%)		Control group (*n* = 212) Number (%)
**Gender**			
Male	69 (35.2)		43 (20.3)
Female	127 (64.8)		169 (79.7)
**Age (Years)**			
Below 20	0 (0.0)		0 (0.0)
20–29	54 (27.6)		25 (11.8)
30–39	62 (31.6)		99 (46.7)
40–49	56 (28.6)		58 (27.4)
50–59	21 (10.7)		28 (13.2)
60 or above	3 (1.5)		2 (0.9)
**Teaching Experience (Years)**			
Below 5	46 (23.5)		22 (10.4)
5–9	37 (18.9)		40 (18.9)
10–14	24 (12.2)		56 (26.4)
15–19	37 (18.9)		41 (19.3)
20–24	30 (15.3)		33 (15.6)
25–29	12 (6.1)		9 (4.2)
30–34	5 (2.6)		10 (4.7)
35–39	4 (2.0)		1 (0.5)
40–44	0 (0.0)		0 (0.0)
45–49	1 (0.5)		0 (0.0)
**School Type** *			
Primary School	73 (37.2)		110 (51.9)
Secondary School	86 (43.9)		65 (30.7)
Special School	37 (18.9)		37 (17.5)
**Received First Aid Training**			
Yes	115 (58.7)		109 (51.4)
No	81 (41.3)		103 (48.6)
**Learnt Dental Injury Management in First Aid Training**			
Yes		25 (12.8)	20 (9.4)
No		171 (87.2)	192 (90.6)
**Confident in Distinguishing Type of Teeth (Deciduous vs Permanent)**			
Yes		43 (21.9)	53 (25.0)
No		153 (78.1)	159 (75.0)
**Read or Heard Dental Injury Information Outside of First Aid Training**			
Yes		19 (9.7)	20 (9.4)
No		177 (90.3)	192 (90.6)

* This is the type of school the teacher was working at when filling out the questionnaires. For the school with both a primary and secondary section, as there was no information on the section he/she was working at, we used the school type he/she worked longer at.

No statistical test for comparison of baseline for both groups [27].

The scores of each question in both questionnaires, along with the difference in score of the two questionnaires for each group, are listed in Table 1.

The result of the multiple linear regression is illustrated in Table 3. The covariates included in the final model were: group (intervention/control), gender, age group, school, acquisition of dental knowledge from other sources, baseline score, and the interaction between group and acquisition of dental knowledge from other sources.

**Table 3:** Relationship between score change and intervention/demographics/characteristics.

	Estimate	95% Confidence Limits		p-value
**Intercept**	−0.1357	−1.500	1.2283	0.8456
**Group** * **(Intervention = 1, Control = 0)**	2.6656	2.0345	3.2967	<0.0001
**Gender (Male = 0, Female = 1)**	0.6449	−0.0101	1.2999	0.0544
**Age Group** # *	−0.3928	−0.6913	−0.0943	0.0103
**School Type**				
**- Primary vs. Special** *	0.9683	0.1486	1.7880	0.0253
**- Secondary vs. Special** *	1.1103	0.2718	1.9488	0.0123
**Prior acquisition of dental knowledge from** **other sources (Yes = 1, No = 0)** *	1.8776	0.5238	3.2314	0.0068
**Baseline Score** *	−0.3091	−0.4104	−0.2078	<0.0001
**Group x Prior acquisition of dental knowledge from other sources** *	−2.7132	−4.6460	−0.7804	0.0062

Estimated ICC = 0.00296.

* The independent variable is significantly different from zero at 5% significance.

# Age Group = 1 if age is less than 20 years, Age Group = 2 if age is between 21 and 30 years, and so forth.

From the regression analysis, the interaction between the two variables, namely group and the acquisition of dental knowledge from other sources, was significant. The regression analysis indicates that the intervention had a significant effect on the score change; this effect is different for those who had and had not acquired dental knowledge. For individuals who had not acquired dental knowledge from sources other than first-aid training, those in the intervention group show significantly larger score improvement (with an average of score increase of 2.6656, p-value <0.0001) in the second questionnaire compared to the improvement in the control group. For individuals who had acquired dental knowledge from sources other than first-aid training, the score change for those who were in the intervention group was 0.0476 lower (p-value = 0.96) than the score change for those in the control group on average, but the difference is insignificant.

To give a rough estimate of the overall effect of the posters on the population, we calculated the average effect of the intervention on people who had and had not acquired dental knowledge, weighted according to the sample proportions. Since the proportion of people who had not acquired dental knowledge was 0.9044, the overall effect of the poster is an average score increase of 2.4063 (p-value <0.0001). Therefore, the overall effect of the intervention is significant.

The estimated intra-cluster correlation coefficient (ICC) was also calculated from the observed data for reference for future research with similar settings. Estimation of the ICC of the questionnaire was performed using the pooled sample with the pre-intervention data. The estimate is calculated using the standard estimator defined by Donner [28]; the value was 0.05128, which was within the expected range.

## Discussion

The effectiveness of educational posters on knowledge regarding management of dental trauma among primary and secondary school teachers was studied. Displaying educational posters for 2 weeks improved the score statistically significantly for those who had not acquired dental knowledge from sources besides first aid training (with an average score increase of 2.6656, p-value <0.0001).

This is the first cluster randomised controlled trial for investigating the effectiveness of educational posters on dental trauma. The results reflect the combination of possibilities with respect to how teachers read, understood, and remembered the poster(s), and are similar to those of the poster study by Lieger et al [21] discussed earlier.

Teachers working in primary or secondary schools in Hong Kong who can read Chinese or English were included in this study. There were 1341 primary and secondary schools (including those with both primary and secondary sections in the same school and schools for children with disabilities) in Hong Kong with a total of 53444 teachers in 2011–2012. Chinese and English are the two official languages in Hong Kong, so the results of the study results apply to all 53444 teachers, who taught 789968 students in Hong Kong.

The inclusion criteria were primary and secondary school teachers who read Chinese and English, so the results will likely be able to be generalised to other countries given that the posters are written in those countries’ official languages for teachers with similar backgrounds and school settings. However, whether or not one reads an educational poster is dependent on many factors, including an individual’s health consciousness, the degree to which a teacher believes that students may require this care, the experience of dental trauma previously (teacher’s own experience, friends, relatives and students etc), teacher workload, and so forth. The understanding and memorizing of the information related to the ability of the teachers. For these reasons, the degree to which these findings can be generalised is unclear.

As some schools display a lot of information for teachers and change the notices or posters quite frequently, a two-week display of the educational poster was chosen as it was not overly difficult to get schools to comply with this length of time. The information obtained from the Education Bureau was that every primary or secondary school had the 3 places we have previously identified (medical room, staff common room, other notice board for teachers). For this reason, we chose these locations for poster display, ensuring minimal variation in environment for the teachers. Implementation of a research study or educational campaign of at least two weeks is feasible in schools in Hong Kong. Investigation of the long-term effect of these posters is outside the scope of this study, and we suggest that other researchers explore it.

Hank’s balanced salt solution (e.g, Save-A-Tooth), eagle’s medium, ViaSpan, and propolis culture medium were not mentioned in the choices directly in question 14 concerning transportation mediums in the questionnaire because these were not accessible to teachers in Hong Kong. A choice of “others (please specify)” was provided, and teachers could fill these (and other) answers in there if they knew of any of these mediums, but no teacher mentioned any of these solutions.

The randomisation of this trial was blinded to the investigators, data entry staff, and the statistician. Only the secretary learned that group A was the intervention group and group B was the control group, and this information was given to the investigator only after the whole statistical report and the manuscript draft was finished. After that, no information about result or figures had been amended but only the change of group A to intervention group and group B to control group. This method aimed to reduce bias and improve the overall quality of the results.

Educational posters are relatively cheap and easy to distribute, and no time limit exists, as teachers do not need to be gathered (as would, for example, a group of teachers attending a seminar or lecture). The three locations chosen for poster display were practical, and long-term display in a medical office at a school is a feasible option. In summary, this is an effective means of improving teachers’ knowledge of dental trauma.

## Conclusion

The display of educational posters at primary and secondary schools in Hong Kong for 2 weeks significantly improves the level of teachers’ knowledge on the management of dental trauma.

## Supporting Information

Checklist S1 (DOCX)

Protocol S1 (PDF)

Chinese Educational poster S1 (PDF)

English Educational poster S1 (PDF)
