# Supplementary material for: Effectiveness of Educational Poster on Knowledge of Emergency Management of Dental Trauma–Part 1. Cluster Randomised Controlled Trial for Primary and Secondary School Teachers
Source: PLoS One. 2013 Sep 11;8(9):e74833. doi: 10.1371/journal.pone.0074833 (PMC3797909; doi:10.1371/journal.pone.0074833)
Supplement: Chinese Educational poster S1 — (PDF) [file pone.0074833.s003.pdf]

# 牙齒創傷

## 處理方法

**恆齒**被撞脫，應立刻放回原來的位置，但乳齒則不應放回，因為很容易影響下面的恆齒。

分辨乳齒或恆齒並不容易，大約5-12歲期間，兒童開始長出1-28隻恆齒，但有些少年12歲以上仍未換掉所有乳齒。

因此，為了簡單及容易記，請以同樣方法處理，然後由牙醫診治，你的幫助很重要！

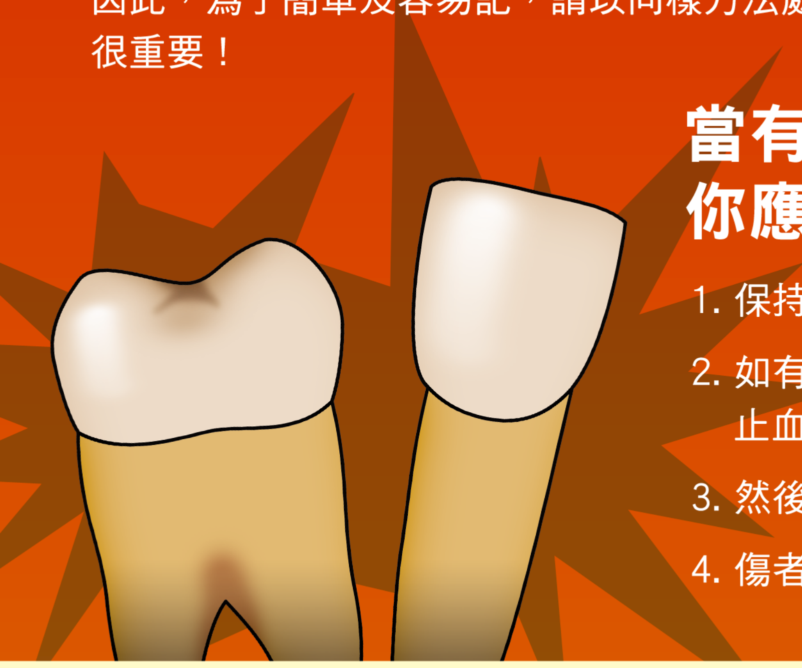

### 當有牙齒創傷的事故，你應該：

1. 保持鎮靜及小心處理。
2. 如有流血，傷者應咬緊紗布或棉花棒止血。
3. 然後根據以下不同的情況處理。
4. 傷者應立即見牙醫。

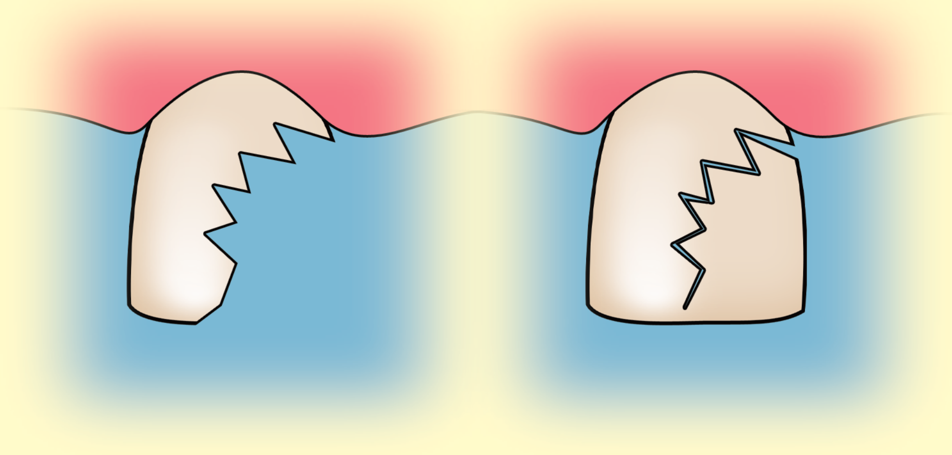

### 牙齒斷裂

- 找尋斷裂的部份，你可能分辨不出斷裂的部份是全隻牙齒或一部份牙齒；抓住牙冠，不應接觸牙腳。
- 立刻把該部份放入注了**生理鹽水（由藥房買來）**，**凍的牛奶**或**傷者的口水**的器皿內，請勿使用其他液體，保持牙腳表面細胞濕潤，以免乾涸死亡，立刻見牙醫。

### 牙齒鬆動或移位：

- 如你曾接受過有關訓練，傷者並不驚慌，你亦有信心，可以嘗試把牙齒移回原來位置，然後立刻見牙醫。
- 要是你不肯定可以做到，應要求**傷者小心慢慢咬合上下排牙齒**，如果移位幅度不是十分大，這樣牙齒會慢慢回到原位。如果移動了的牙齒阻止上下排合上，則顯示幅度太大，未能自然回復原位，應停止，立刻見牙醫。

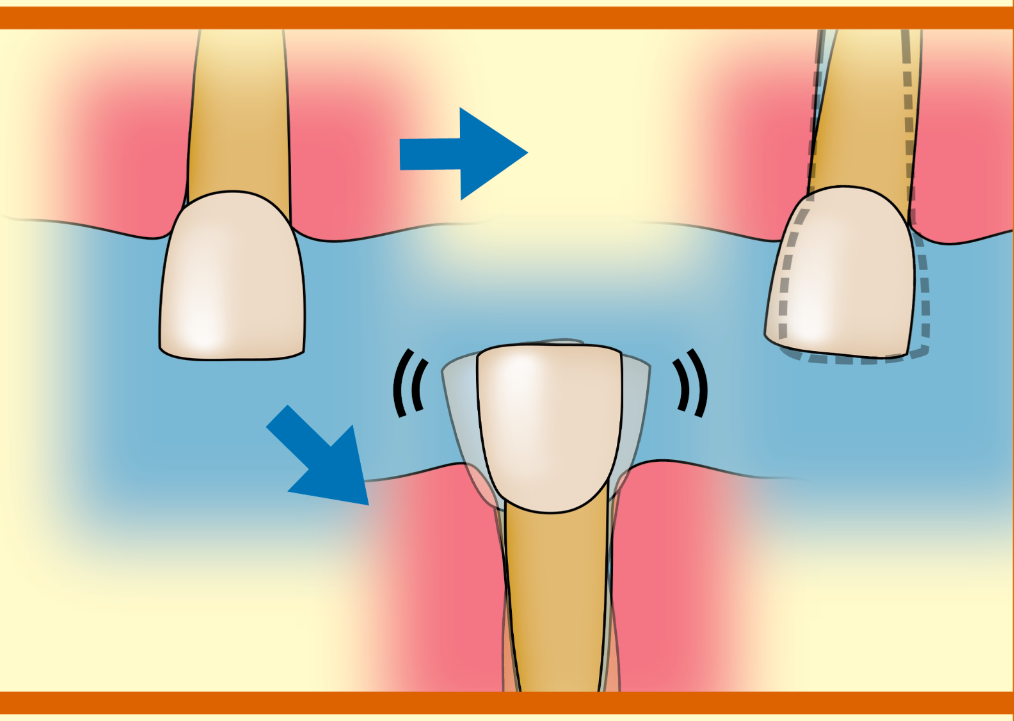

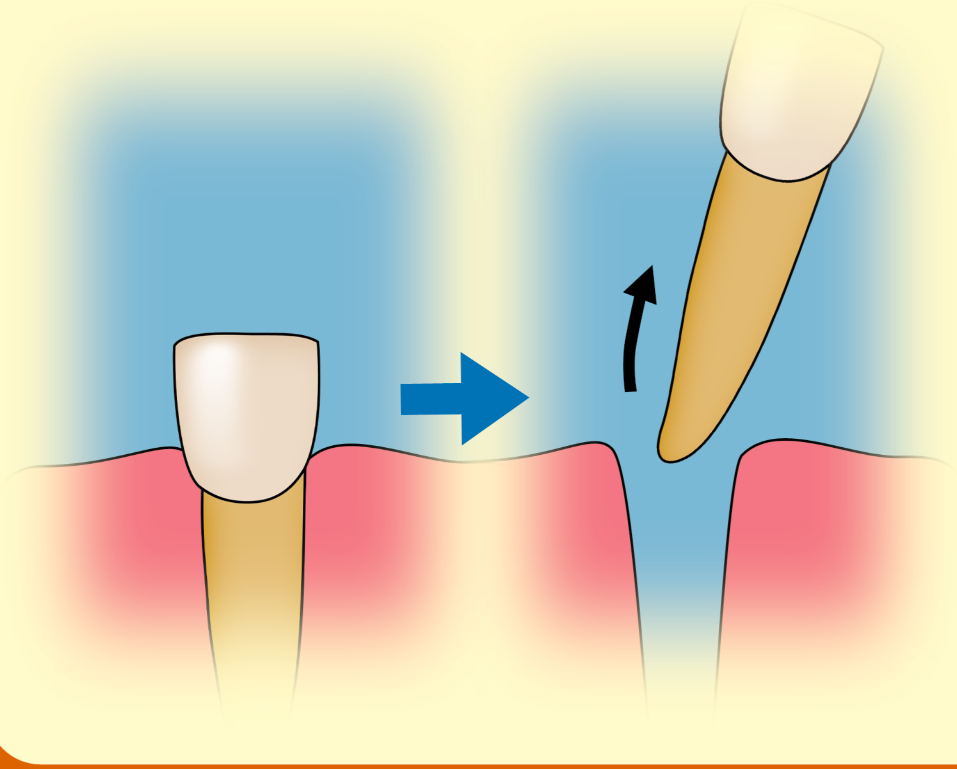

### 撞脫牙齒：

- 小心尋找牙齒，抓住牙冠，不應接觸牙腳。
- 如你曾接受訓練及有信心分辨是恆齒，先塞洗手盤，以自來水沖洗牙齒10秒，然後放回原位。
- 否則，即使牙齒十分骯髒，亦不應清潔牙齒，把牙齒放入注了**生理鹽水（由藥房買來）**，**凍牛奶**或**傷者口水的器皿**，請勿使用其他液體，保持牙腳表面細胞濕潤，以免乾涸死亡。
- 立刻見牙醫。

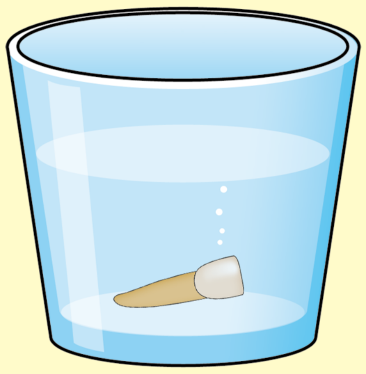

Effectiveness of Educational Poster regarding dental trauma.　Cecilia Young et al
