# Supplementary material for: Effectiveness of Educational Poster on Knowledge of Emergency Management of Dental Trauma–Part 1. Cluster Randomised Controlled Trial for Primary and Secondary School Teachers
Source: PLoS One. 2013 Sep 11;8(9):e74833. doi: 10.1371/journal.pone.0074833 (PMC3797909; doi:10.1371/journal.pone.0074833)
Supplement: English Educational poster S1 — (PDF) [file pone.0074833.s004.pdf]

DENTAL INJURY

guideline to handle

If a **permanent tooth** is knocked out, it could be put back. If a baby tooth is knocked out, it should not be put back. It will harm the underlying permanent tooth.

It is difficult to distinguish between a baby tooth and a permanent tooth. From around aged 5 – 12, children start to have 1 to all 28 permanent teeth. But some teenagers still retain 1 to more baby tooth after 12.

Therefore, make it simple and easy to remember, just deal with them with the same following methods, and let the dentists to treat them. You can help a lot !

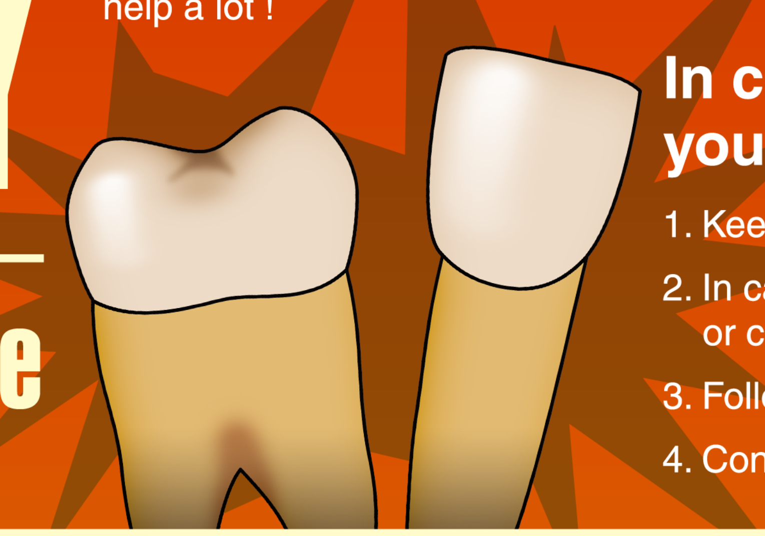

In case of dental injury, you should:

1. Keep calm and handle carefully
2. In case of bleeding, bit on a gauze or cotton roll to stop it.
3. Follow the different situations below
4. Consult a dentist immediately.

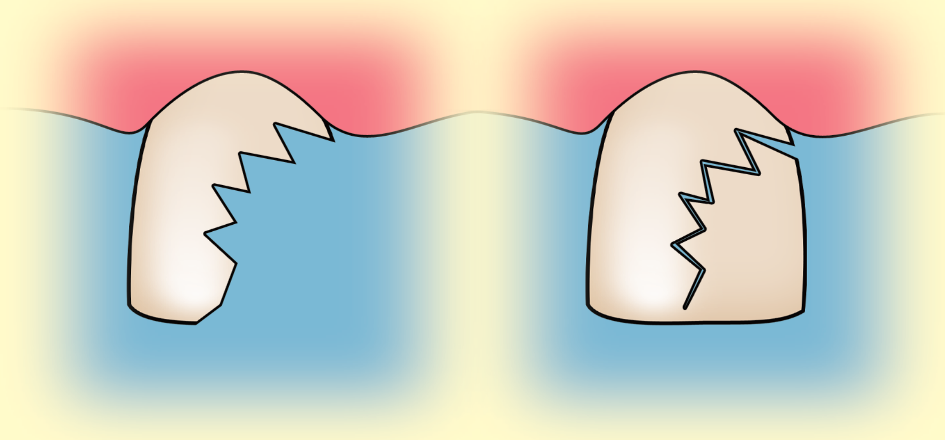

Tooth Fracture

- Look for the fractured piece, you may not distinguish a fractured part of a tooth from a whole tooth, hold the crown, do not touch the root.
- Put it in a container with **physiological saline (from pharmacy)** / **cold milk** / **patient's saliva** at once to keep it moist and alive. Do not use other liquids. Go to dentist immediately.

Mobile or displaced tooth:

- If you have learnt it and still feel confident, put it back to the original position of a calm patient.
- If you are not sure, do not try to move the tooth, only ask the patient to **close the mouth and bring the teeth together carefully and slowly,** the tooth will get back to it's natural position when the position is not too bad. If it is interfered and seems not return to the original position, stop it! Then go to the dentist immediately.

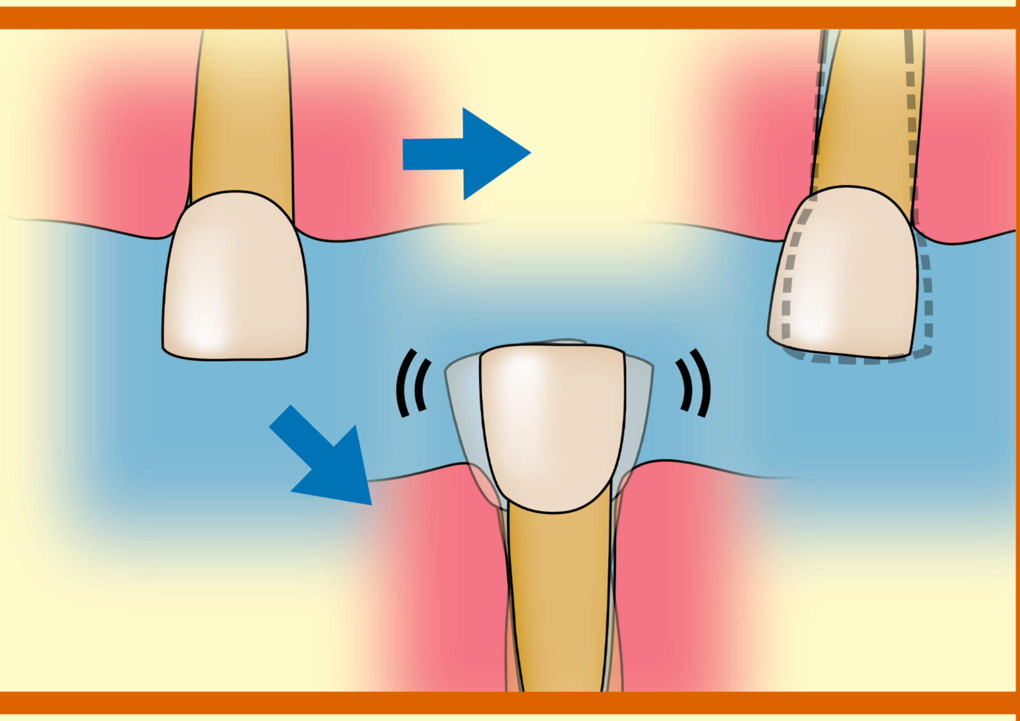

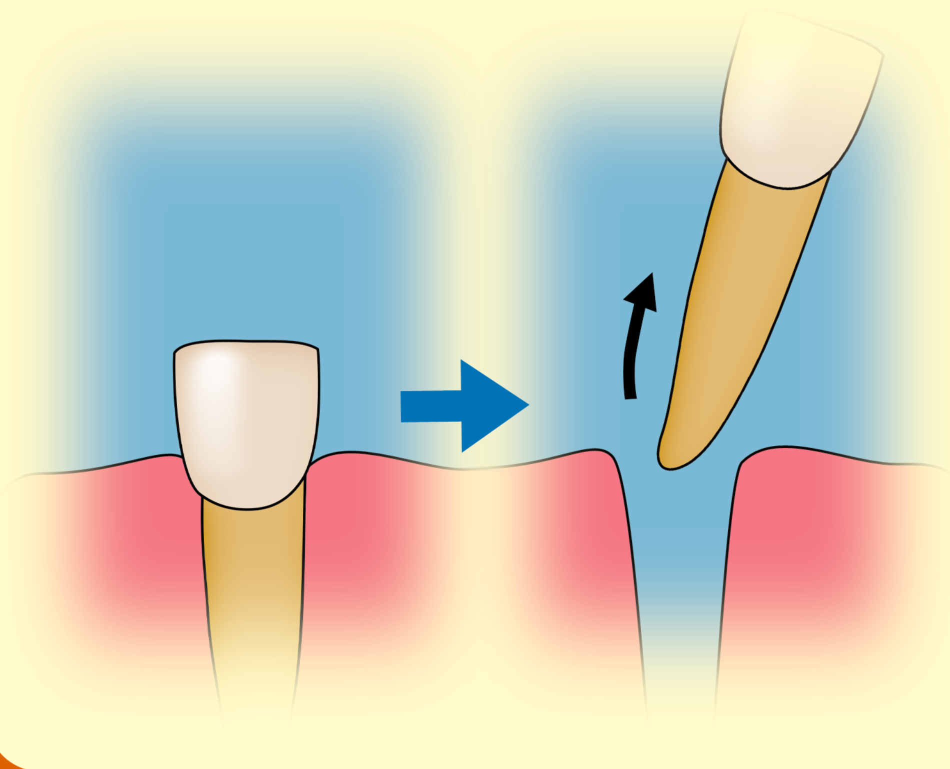

Tooth knocked out:

- Look for the tooth and hold the crown only, do not touch the root.
- If you have learnt the correct procedures before and still feel confident, plug the sink, rinse the tooth in tap water for 10 seconds and put back to the socket.
- Otherwise, do not clean the tooth even it is dirty! Put it in a container with **physiological saline (from pharmacy)**, **cold milk** or **in patient's saliva.** Keep it moist and alive. Do not use other liquids. Then go to the dentist immediately.

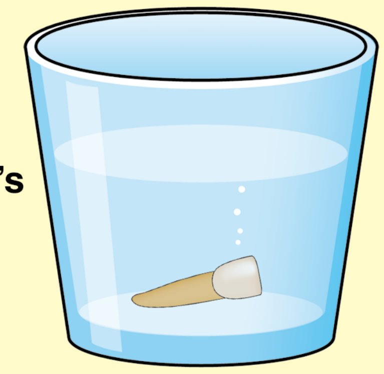

Effectiveness of Educational Poster regarding dental trauma. Cecilia Young et al
