# Supplementary material for: Effectiveness of Educational Poster on Knowledge of Emergency Management of Dental Trauma–Part 1. Cluster Randomised Controlled Trial for Primary and Secondary School Teachers
Source: PLoS One. 2013 Sep 11;8(9):e74833. doi: 10.1371/journal.pone.0074833 (PMC3797909; doi:10.1371/journal.pone.0074833)
Supplement: Protocol S1 — (PDF) [file pone.0074833.s002.pdf]

香港大學

香港大學及醫管局港島西醫院聯網研究倫理委員會

醫院管理局
HOSPITAL
AUTHORITY

Institutional Review Board of the University of Hong Kong/

Hospital Authority Hong Kong West Cluster (HKU/HA HKW IRB)

Address: Rm 901, Administration Block, QMH Tel 2255 3923 2255 4086 Fax 2255 4735

Dr. Cecilia Young
105A, 1/F Liberte Place
833 Lai Chi Kok Rd, Kln

22-Feb-11

Dear Dr. Young,

IRB Reference Number: **UW 11-077**

The HKU/HA HKW IRB is authorized by a joint agreement of the University of Hong Kong and Hospital Authority Hong Kong West Cluster to review and monitor clinical research. It serves to ensure that research complies with the Declaration of Helsinki and acts in accordance to ICH GCP guidelines, local regulations and Hospital Authority and the University policies.

I write to inform that your research application/submission has been approved by an expedited process with details shown below. You are also requested to adhere to the conditions listed.

**Protocol title**	:	Effectiveness of educational poster to primary and secondary teachers regarding emergency management of dental trauma - A cluster randomised controlled trial
**Study site(s)**	:	As stated in application form
**IRB reviewer**	:	Dr. Sydney Tang, Deputy Chairman of the HKU/HA HKW IRB
**Document(s) approved**	:	01. Clinical research ethics review application form
	:	02. Protocol (P01 17.11.2010)
	:	03. Invitation letter (amended 16.2.2011) - English
	:	04. School's informed consent form (amended 16.2.2011) - English and Chinese
	:	05. Teacher's informed consent form (amended 16.2.2011) - English and Chinese
	:	06. Questionnaire - English and Chinese
	:	07. Advertisement - English and Chinese
**Document(s) reviewed**	:	08. Lay knowledge of physical education teachers about the emergency management of dental trauma in Hong Kong Dental Traumatology 2001; 17: 77-85
	:	09. Impact of educational posters on the lay knowledge of school teachers regarding emergency management of dental injuries Dental Traumatology 2009; 25:406-412
	:	10. Short CV of principal investigator

(Conditions : 1. Do not deviate from, or make changes to the study protocol without prior written IRB approval, except when it is necessary to eliminate immediate hazards to research subjects or when the change involves only logistical or administrative issues.

2. Report the following to HKU/HA HKW IRB: (i) study protocol or consent document change (use 'HKU/HA HKW IRB RE001F7'), (ii) serious adverse event (use 'HKU/HA HKW IRB RE001F8'). (iii) study progress (use 'HKU/HA HKW IRB RE001F9a') (iv) new information that may be relevant to a subject's willingness to continue participation in the study.

3. Report study progress to HKU/HA HKW IRB at a 12-monthly interval until study closure.)

Yours sincerely,

W. H. Lee
HKU/HA HKW IRB Secretary

Protocol

Effectiveness of educational poster to primary and secondary teachers regarding emergency management of dental trauma - A cluster randomised controlled trial

Background

Teachers face many students. In an accident, if they have the knowledge to take simple but essential emergency steps before the students reach the professional care, they help the saving of the injured teeth.

Physical education teachers in Hong Kong have inadequate knowledge even most of them received first aid training (1). This study was performed around 2000, there was no education given to the PE teachers. Since no other related education has been given to primary and secondary education in these 10 years, therefore, it is predicted that most of teachers in Hong Kong have inadequate knowledge about emergency care about dental injury.

Poster education was performed in Switzerland and shown effective (2). In the study posters were sent to 155 schools in Canton of Bern in 2001, after 5 years, 1000 questionnaires were sent to schools in that area and compare with 1000 teachers in another area. Since there is no baseline information in the study in Switzerland, a cluster randomized controlled trial is planned to investigate the effectiveness of the educational poster.

Aim:

To investigate the effectiveness of the educational poster about dental injuries.

Methodology:

Design: We conduct a cluster randomized controlled trial. Lists of Primary and secondary schools were requested from the Education Bereau. Schools will be invited to join the study and then randomized to the intervention group and the control group in the unit of school using computer generated numbers. All teachers will fill in the first questionnaire and send it back to the investigator in 1 week. Then 3 copies of the same poster will be given to the intervention schools, these schools will be requested

to place them in 1. the medical room 2. staff common room and 3 anywhere that information is usually placed for the teachers. No poster will be given to the control group. After 2 weeks, the poster will be removed. The same questionnaire will be filled in by all the teachers in both groups and sent back in 1 week. Then the data will be processed to show whether the poster is effective to improve the knowledge of the teachers.

This study will comply with Declaration of Helsinki and ICH GCP guidelines.

Eligibility

:

All primary and secondary schools in Hong Kong that the teachers can read Chinese or English.

Sample size calculation:

In determining the k, the number of cluster required to achieve a power of (1-β) and a significance of α, we consider the following formula (3):

$$k = \frac{\left(z_{\alpha/2} + z_{\beta}\right)^2 (2\sigma^2)[1 + (m-1)\rho]}{m(\mu_1 - \mu_2)^2}$$

In this formula, $z_{\alpha/2}$ and z_{β} are standard normal distribution values corresponding to upper tail probabilities of $\alpha/2$ and β respectively. m is the cluster size, ρ is the intracluster correlation coefficient. $|\mu_1 - \mu_2|$ is the minimum difference in outcomes of intervention and control groups which can be regarded as significant, and σ is the standard deviation of the outcome variable.

The outcome variable is the score change in the questionnaire before and after the study. In order to demonstrate a difference in score change of 2 marks (variance 10) between the intervention group and the control group, with a power of 90% and a statistical significance of 5%, 53 individuals are needed in each group under simple random sampling. To account for the cluster design, we assume an intracluster correlation (ICC) of 0.05. No published data on ICC under this setting can be found. However, in general practice studies, ICC take values commonly between 0.01 and 0.05 (4). With an average of not exceeding 40 teachers recruited per school, the adjusted sample size is 156 individuals per group. To allow for potential dropouts, we

aimed to recruit extra 30% individuals per group, yielding a total of 203 individuals per group. Assume an average of 25 teachers per school can be recruited, 9 schools per group are needed. The number of schools will be adjusted accordingly to the number of teachers participating per school, to reach a total of more than 203 teachers. A minimum of 6 schools will be recruited per group.

To obtain a better estimate for the variance, we will conduct a pilot test with two schools (with about 60 teachers). In the pilot test, we ask each teacher to fill out the questionnaire once. We estimate the variance of the difference in score change between the intervention group and the control group by two times the sample variance of the scores obtained from the pilot test. The sample size will then be adjusted according to the new estimated variance according to the pilot test.

Recruitment:

We send invitation letters with school consent form and teacher consent form to lots of 50 schools randomly from the schools in the above-mentioned school lists. We aimed at 12 - 18 schools, as we expect the average number of teachers participating per school will be from 25 to 40. We will then randomize them to the intervention group and control group in the unit of school. The sample size will be finalized after the pilot test from 2 randomly schools first, one for control and one for intervention

Withdrawal of teachers from the study

The participating teachers can withdraw from the study at any time.

Statistical Analysis

Only teachers who have completed both questionnaires before and after the study will be included in the analysis. To accommodate possible dependence between observations from the same school, multilevel regression will be conducted to investigate the changes of questionnaire score before and after the study. In the regression model, the score change will be considered as the dependent variable. The group (intervention/control), baseline questionnaire score, institution (primary/secondary school), gender, first-aid training experience (yes/no), and interaction terms between group and institution, gender and first-aid training experience are considered as potential factors that will be included in the analysis.

Significance of the regression coefficients will be tested at 5% level of significance. All tests are two-sided.

Ref:

1. Lay knowledge of physical education teachers about the emergency management of dental trauma in Hong KongDental Traumatology 2001; 17: 77–85
2. Impact of educational posters on the lay knowledge of school teachers regardingemergency management of dental injuries Dental Traumatology 2009; 25: 406–412;
3. Allan Donner and Neil Klar. Design and Analysis of Cluster Randomization Trials in Health Research. London: Arnold, 2000.
4. Underwood M, Barnett A, Hajioff S. Cluster randomization: a trap for the unwary. Br J Gen Pract 1998; 48: 1089-1090.

Name: **first questionnaire**

School:

Date:

Effectiveness of educational poster to primary and secondary teachers regarding emergency management of dental trauma - A cluster randomised controlled trial

Part 1 demographics

1. Gender: ○a. Male ○b. female

2. Age: ○a. under 20 ○b. 20-29 ○c. 30-39
   ○d. 40-49 ○e. 50 or above

3. Working place and duration (also give the part time/ voluntary job)
   ○a. Primary School ______ years ○b. Secondary schools _____ years
   ○c. others (please specify) ______ years

4. Have you received any formal first aid training?
   ○a. Yes (go to Q5) ○b. No (go to Q6)

5. Is the first aid about dental injury included in your formal first aid training?
   ○a. Yes ○b. No

6. Do you think that you can distinguish a primary tooth (baby tooth) from a permanent tooth by considering the patient's age and looking at the tooth, the socket or the position?
   ○a. Yes ○b. No

7. Have you ever received any educational material/information about the injury except formal first aid training (before this study)?
   ○a. Yes (go to Q.8) ○b. No (go to Q.9)

8. Which way?
   ○a. TV ○b. Journals ○c. Radio ○d. School
   ○e. Poster ○f. Newspapers ○g. Internet ○h. Oral description
   ○i. Others (please specify) ____________________

Part II knowledge

9. If there is a dental injury, the impact site is the teeth, only the teeth and surrounding part seemed to be injured, what should the patient do?

- ○ a. Go to the casualty in the nearest hospital on foot or by any transport
- ○ b. Call an ambulance; go to the casualty in the nearest hospital.
- ○ c. Go to the nearest private doctor
- ○ d. Go to the patient's family doctor
- ○ e. A dentist
- ○ f. Treat it by self
- ○ g. Others (please specify)____________________
- ○ h. Don't Know

10. When should the patient go or be brought for the examination and treatment?

- ○ a. At lunch or after school
- ○ b. After plenty of rest
- ○ c. Within 24 hours
- ○ d. Within 48 hours
- ○ e. When the parent or guardian is free to bring the patient for examination and treatment
- ○ f. Any time when the patient feels relax and want to have treatment
- ○ g. Within 4 hours
- ○ h. Immediately
- ○ i. Others (please specify) ____________________
- ○ j. Don't Know

11. If the tooth is fractured, the patient or surrounding person should:

- ○ a. The fractured part is useless, ignore it
- ○ b. Try to find the fractured part, wrap it with gauze or tissue and bring it for examination and treatment
- ○ c. Put it in liquid medium and bring it for examination and treatment
- ○ d. Others (please specify) ____________________
- ○ e. Don't Know

12. If the tooth is displaced or extruded but still attached, the patient or surrounding person should:

- ○ a. Do not touch, let it remains its new position
- ○ b. Try to put back to the original position
- ○ c. Ask the patient to carefully clench one's teeth if it is possible
- ○ d. Others (please specify) ____________________
- ○ e.. Don't Know

13. Should a knocked out tooth be put back to the original position?

Baby tooth	○a. Yes	○b. No	c.○Don't know
Permanent tooth	○a. Yes	○b. No	c.○Don't know

14. If a tooth is knocked out? It should be: (1 or more)

- ○ a. The tooth is useless, do not spend time to find it or to work on it
- ○ b. Wrap it in gauze or tissue and bring it to examination and treatment
- ○ c. Soak it in cold milk in a container and bring it to examination and treatment
- ○ d. Soak it in physiological saline in a container (from pharmacy) and bring it to examination and treatment
- ○ e. Soak it in the patient's saliva in a container and then bring the container to examination and treatment
- ○ f. Soak it in tap water in a container and bring the container to examination and to treatment
- ○ g. Soak it in distilled water in a container and bring the container to examination and to treatment
- ○ h. Put it in a container or a plastic bag in dry condition and bring it to examination and treatment
- ○ i. Soak it in a container with disinfectant solution and bring it to examination and treatment
- ○ j. Others (please specify) ____________________
- ○ k.. Don't Know

End of the questionnaire

老師姓名(正楷) **第一份問卷**

學校: ______________________________

日期

牙齒創傷教育性海報提高中小學老師知識的效能-群組隨機對照試驗

第一部份. 基本統計資料

請在適當橫線上填上✓號。

1. 性別 ： ○ a.男 ○ b.女

2. 年齡 ： ○ a.20 以下 ○ b.20-29 ○ c.30-39
○ d.40-49 ○ e.50-59 ○ f.60 或以上

3. 工作地點及年數（請同時填上正職以外的工作，如：列明兼職或義務工作）：
○ a.小學 ___年 ○ b.中學 ___年 ○ c.其他（請列明）___年

4. 你曾否接受過正規急救訓練？
○ a.有 （ 往第 5 條 ） ○ b.沒有 （ 往第 6 條 ）

5. 正規急救課程內容有否包括牙齒創傷？
○ a.有 ○ b.沒有

6. 你認為自己是否能從傷者年齡，觀察受傷牙齒外形、牙床及其位置分辨出乳齒或恆齒？
○ a.是 ○ b.不是

7. 除正式急救外，在這次調查以前，你曾否閱讀或收聽過任何有關牙齒創傷的教育資料？
○ a.有 （ 往第 8 條 ） ○ b.沒有 （ 往第 9 條 ）

8. 你從哪種途徑得悉有關牙齒創傷的教育資料？
○ a.電視 ○ b.雜誌 ○ c.電台 ○ d.學校
○ e.海報 ○ f.報紙 ○ g.互聯網 ○ h.口頭描述
○ i.其他（請列明）____________________

第二部份 (有關知識)

9. 如果有意外，以致牙齒創傷，其受傷位置只在牙齒及附近口腔位置，傷者應怎樣做？

○ a. 步行或以交通工具往最近的醫院急症室
○ b. 召救傷車，往最近醫院急症室
○ c. 往最近的私人執業全科醫生
○ d. 往傷者的家庭醫生
○ e. 往牙醫
○ f. 自行處理
○ g. 其他（請列明）＿＿＿＿＿＿＿＿
○ h. 不知道

10. 傷者應何時前往或被送往診治？

○ a. 午膳或放學後
○ b. 充分休息後
○ c. 二十四小時內
○ d. 四十八小時內
○ e. 當家長或監護人有空帶傷者前往診治時
○ f. 當傷者放鬆心情並希望診治時
○ g. 四小時內
○ h. 立刻
○ i. 其他（請列明）＿＿＿＿＿＿＿＿
○ j. 不知道

11. 如牙齒斷裂，傷者或附近人士應：

○ a. 斷裂部份無用，不需理會
○ b. 找出斷裂部份，以紗布或紙巾包裹帶往診治
○ c. 把斷裂部份放入液體內，帶往診治
○ d. 其他（請列明）＿＿＿＿＿＿＿＿
○ e. 不知道

12. 如牙齒依然留在牙床內但移位或拉出了一些，傷者或附近人士應：

○ a. 不應接觸，保持現在新的位置
○ b. 嘗試用手指把它移回原位
○ c. 如有可能，請傷者小心慢慢地咬合
○ d. 其他（請列明）____________________
○ e. 不知道

13. 碰撞後飛脫的牙齒是否應該放回牙床原來位置？

乳齒	a ○應該	b. ○不應該	c.○不知道
恆齒	a ○應該	b. ○不應該	c.○不知道

14. 如牙齒碰撞後飛脫，應：(可選擇一個或以上)

○ a. 牙齒沒有用處，不應浪費時間找尋及處理
○ b. 以紗布或紙巾包裹，帶往診治
○ c. 放入凍牛奶內，浸過牙齒，帶往診治
○ d. 放入生理鹽水內（從藥房買來的），浸過牙齒，帶往診治
○ e. 放入盛有傷者的口水的器皿內，浸過牙齒，帶往診治
○ f. 放入盛有自來水的器皿內，浸過牙齒，帶往診治
○ g. 放入盛有蒸餾水的器皿內，浸過牙齒，帶往診治
○ h. 放入器皿或膠袋內，乾的狀態下，帶往診治
○ i. 放入盛有消毒藥水的器皿內，浸過牙齒，帶往診治
○ j. 其他（請列明）____________________
○ k. 不知道

問卷完

親愛的校長

醫學研究 — 牙齒創傷

香港大學的張念光教授及黃堅祐先生與我現進行牙齒創傷的醫學研究，我們邀請貴校參與。(請參考 ethical approval.jpg)

參與學校是全港第一批接觸有關牙齒創傷即時處理方法的學校，內容包括傷者於正式醫治前簡單而必需的處理步驟，正確方法能救回更多牙齒。

是次研究目的為測試教育性海報能否提高老師牙齒創傷方面的知識，只需**大約十分鐘閱讀**，以調查資料能否提高老師的知識。完成後，全港所有中小學均會收到相同教育性海報，印制及寄出海報需以此研究結果為基礎，貴校的參與對所有中小學十分重要。

參與老師需填二份選擇題問卷，有十四條選擇題，**大約需要五分鐘**，內容為基本資料及牙齒創傷問題，不涉及敏感問題，並會依從所有香港港法例及國際性的醫學研究倫理守則（包括保密及其他）。

請參與本研究，參與老師需填二份選擇題問卷，研究人員需要二份結果用作分析。**但參與老師即使已簽署同意書仍有絕對權利退出研究。**

我們曾把問卷及海報給予一些中學生及其監護人，他們均認為牙齒創傷資料對日常生活有用，希望研究完成，有基礎印制及寄出此海報，全港中小學均能接觸有關牙齒創傷的即時處理方法。

我們已經把所有文件連同回郵信封寄給包括貴校各隨機抽樣的中小學，完成研究後，我們會在寄出海報給各中小學時，多謝參與的學校，如個別參與學校不希望出現在鳴謝名單，敬請提出。

我們需要您的支持! 請參與是次調查。如有查詢請電 23618684 楊小姐，敬希以回郵信封寄回**學校同意書**及**老師同意書**。謝謝！

研究團隊

Dear Principal and teachers,

Ref: Academic Study for dental injuries – questionnaire

I am a public health practitioner and researcher, I am now co-operating with Prof Cheung Lim Kwong and Mr Alex Wong in an academic study for dental injuries. This is a joint project with the University of Hong Kong. We invite your school to join this study.

Participating schools will be the first group to learn the essential but simple and precise steps of how to help students to deal with dental injuries in accidents before professional care are provided.

The result of this study will give the dental field some information about whether the education for primary and secondary teachers about dental injury improves teacher's knowledge. Also, all primary and secondary schools in Hong Kong will ultimately receive the same education if the result is positive.

Participating schools will receive altogether 2 sets of questionnaire which consist of 14 MC questions. Teachers need to fill the 2 sets of questionnaire. There will be no sensitive questions and all information will comply with rules in Hong Kong and international medical research ethics code.

Please sign the school consent form and individual teacher's consent form if you are willing to help. Completion of 2 sets of questionnaires is necessary to give the data for the analysis. **However, teachers have the absolute right to withdraw at any time even they signed the consent form.**

Please discuss with your staff and sign the consent form if you are willing to participate this study. Kindly contact Dr Cecilia Young at 23618684 if you have any enquiries. Please mail the consent form with the return envelope.

Thank you for your kind attention.

Cecilia Young
Principal investigator

School's Informed Consent Form

Effectiveness of educational poster to primary and secondary teachers regarding emergency management of dental trauma - A cluster randomised controlled trial

1. There will be 2 sets of questionnaires consists of multiple choice questions. Participating teacher needs to complete both of them for data analysis.

2. Education will be given to the teachers. There will be no need of gathering for this.

3. There will be no sensitive questions and all information will comply with all rules in Hong Kong (keep confidential and other law) and international medical research ethics code.

4. The data are used for academic study for whether education to teachers improves teachers' knowledge. It gives the dental field some information. All primary and secondary school teachers will receive the same education if the result is positive.

5. **Participating teacher has the absolute right to withdraw this study at any stage.**

6. A copy of this consent form will be given to the school.

Participating school ______________________________

Tel:

Date:

Person in charge:

____

Authorized signature with school chop

校方知情同意書

牙齒創傷教育性海報提高中小學老師知識的效能-群組隨機對照試驗

1. 參與老師需填二份選擇題問卷，研究人員需要二份結果用作分析。

2. 研究人員會提供牙齒創傷資料，校方不需聚集老師。

3. 問卷內容不涉及敏感問題，並會依從所有香港港法例及國際性的醫學研究倫理守則（包括保密及其他）。

4. 結果用作學術研究用途以調查所提供的資料能否提高老師的知識。本研究將給予牙醫學界一些數據，如牙齒創傷資料能有效傳達知識，全港所有中小學均將會收到相同資料。

5. 參與老師即使已簽署同意書仍有絕對權利退出研究。

6. 本同意書副本將給予校方。

參與學校：______________________________

電話　　：______________

日期　　：______________

負責人　：______________

____

校方蓋印

Teacher's informed Consent Form **(amended 16.2.2011)**

Effectiveness of educational poster to primary and secondary teachers regarding emergency management of dental trauma - A cluster randomised controlled trial

1. There will be 2 sets of questionnaires consists of multiple choice questions. Participating teacher needs to complete both of them for data analysis.
2. Education will be given to the teachers. There will be no need of gathering for this.
3. There will be no sensitive questions. All information will comply with all rules in Hong Kong (keep confidential and other law) and international medical research ethics code.
4. The data are used for academic study for whether education to teachers improves teacher's knowledge. It gives the dental field some information. All primary and secondary school teachers will receive the same education if the result is positive
5. **Participating teacher has the absolute right to withdraw this study at any stage even this consent form is signed.**
6. A copy of consent form will be given to individual participating teacher.

Participating teacher:

I can read Chinese / English questionnaire and information. (Please cross out the one that you cannot read.)

School: ______________________________

Teacher's Print Name:

Date:

____

Signature

參與老師知情同意書 (amended 16.2.2011)

牙齒創傷教育性海報提高中小學老師知識的效能-群組隨機對照試驗

1. 參與老師需填二份選擇題問卷，研究人員需要二份結果用作分析。

2. 研究人員會提供牙齒創傷資料，校方不需聚集老師。

3. 問卷內容不涉及敏感問題，並會依從所有香港港法例及國際性的醫學研究倫理守則（包括保密及其他）。

4. 結果用作學術研究用途以調查所提供的資料能否提高老師的知識。本研究將給予牙醫學界一些數據，如牙齒創傷資料能有效傳達知識，全港所有中小學均將會收到相同資料。

5. 參與老師即使已簽署同意書仍有絕對權利退出研究。

6. 本同意書副本將給予參與老師。

本人能閱讀 中文/英文 問卷及牙科資料。(請刪去不適用語言)

學校: ______________________________

參與老師姓名(正楷):

日期:

____

簽署
